# Comparisons of Physicochemical Properties, Bacterial Diversities, Isoflavone Profiles and Antioxidant Activities on Household and Commercial *doenjang*

**DOI:** 10.3390/molecules28083516

**Published:** 2023-04-16

**Authors:** Hee Yul Lee, Du Yong Cho, Jea Gack Jung, Min Ju Kim, Jong Bin Jeong, Ji Ho Lee, Ga Young Lee, Mu Yeun Jang, Jin Hwan Lee, Md Azizul Haque, Kye Man Cho

**Affiliations:** 1 Department of Green Bio Science and Agri-Food Bio Convergence Institute, Gyeongsang National University, Jinju 52725, Republic of Korea; wjdald99@nate.com (H.Y.L.);; 2 Department of Food Science, Gyeongsang National University, Naedongro 139-8, Jinju 52849, Gyeongnam, Republic of Korea; 3 Department of Life Resource Industry, Dong-A University, 37, Nakdong-daero 550 beon-gil, Saha-gu, Busan 49315, Republic of Korea; 4 Department of Biochemistry and Molecular Biology, Hajee Mohammad Danesh Science and Technology University, Dinajpur 5200, Bangladesh

**Keywords:** *doenjang*, physicochemical, bacterial diversity, isoflavone, antioxidant activity

## Abstract

In this study, the physicochemical properties (pH, acidity, salinity, and soluble protein), bacterial diversities, isoflavone contents, and antioxidant activities of *doenjang* (fermented soy paste), household *doenjang* (HDJ), and commercial *doenjang* (CDJ), were assessed and compared. The values of pH 5.14–5.94 and acidity 1.36–3.03%, indicated a similar level in all doenjang. The salinity was high in CDJ at 12.8–14.6%, and the protein contents (25.69–37.54 mg/g) were generally high in HDJ. Forty-three species were identified from the HDJ and CDJ. The main species were verified to be *Bacillus amyloliquefaciens* (*B. amyloliquefaciens*), *B*. *amyloliquefaciens* subsp. *plantarum*, *Bacillus licheniformis*, *Bacillus* sp. and *Bacillus subtilis*. Comparing the ratios of isoflavone types, the HDJ has an aglycone ratio of >80%, and 3HDJ indicates a ratio of isoflavone to aglycone of 100%. In the CDJ, except 4CDJ, glycosides account for a high proportion of more than 50%. The results of antioxidant activities and DNA protection effects were variedly confirmed regardless of HDJs and CDJs. Through these results, it is judged that HDJs have a variety of bacterial species compared to CDJs, and these are biologically active and converted from glycoside to aglycone. Bacterial distribution and isoflavone contents could be used as basic data.

## 1. Introduction

Soybeans have been used for the long term in East-Asian countries as one of the world’s three most extensive food resources. They supplement nutrients in rice-centered diets, possessing a substantial amount of protein, essential fatty acids, organic acids, minerals, and vitamins generally lacking in grains. Some physiologically active substances, including isoflavone, polyphenol, flavonoid, dietary fiber, soy oligosaccharide, saponin, and lecithin are more greatly found in soybeans than in the other grains, which showed anti-aging, anti-cancer, anti-arteriosclerosis, and hypoglycemic effects [1,2,3]. The raw soybean materials enriched with antioxidant compounds are further converted into different functional metabolites after heating, germination, and fermentation [4,5]. Notably, the isoflavone aglycones, such as daidzein, glycitein, and genistein, were extracted from the isoflavone glycosides of soybean during fermentation [6]. Moreover, phenolic compounds such as vanillic acid, chlorogenic acid, *p*-coumaric acid, ferulic acid, caffeic acid, and saponins have also been reported from soybeans [7,8,9].

The traditional fermented food *doenjang* has been accepted by consumers owing to its excellent level of amino acids, essential fatty acids, and phytoestrogens that belong to the major ingredients such as soybean with *Cheonggukjang* and soy sauce [10,11,12]. According to the manufacturing technique, the soybean pastes are divided into traditional and improved soybean pastes. The preparation of *doenjang* takes about six months for its processing, where *meju* is the major ingredient. *Meju* is made of fermented soybeans without adding starter cultures. During *doenjang* fermentation, bacteria and fungi from the source materials (rice straws) and environment (air), etc., are fastened to *meju* [13]. Therefore, *meju* contributes to divergent microorganisms that are directly involved in the fermentation of *doenjang* [14,15]. Diverse bacterial and fungal communities, including an abundance of *Tetragenococcus*, *Bacillus*, *Chromohalobacter*, *Aspergillus,* and *Debaryomyces,* were observed in traditional Korean *doenjang* [16]. In fact, the traditional *doenjang’s* filtered liquid (soy sauce) qualities were influenced by different microorganisms during aging [17,18]. In contrast, commercial *doenjang*, compared to traditional *doenjang*, needs a short time and is made with koji, soybean, and salt. Koji was made by inoculating wheat, rice, and barley with *A. oryzae* [17,18,19,20]. For this reason, the *doenjang* shows differences in microorganisms based on the manufacturing method and materials.

Till date, the differences in microbial diversity, physicochemical qualities, isoflavone contents, and antioxidant activities of the household *doenjang* (HDJ) and commercial *doenjang* (CDJ) were not reported yet. Four household *doenjang* (HDJ) samples and four commercial *doenjang* (CDJ) samples obtained from several locations were studied. The physicochemical features, antioxidant activities, secondary metabolites (isoflavone), and the diversity of cultured microorganisms (based on the rRNA) were analyzed. These results will suggest various indicators for comparing the quality of the HDJ and CDJ.

## 2. Results and Discussion

### 2.1. Physicochemical Properties of HDJs and CDJs

The differences in the physicochemical qualities of HDJ and CDJ are shown in Table 1. The pH value ranged from 5.31–5.94 in HDJs, but 5.14–5.64 was obtained in CDJs. The acidity was also 1.36–3.03% in HDJs and 1.59–2.87% in CDJs. Among these, the 4HDJ demonstrated the highest acidity of 3.03%. There was no significant difference among these, and the highest pH was 5.94 in 4HDJ. Obviously, no significant differences in pHs and acidities were observed among the HDJ and CDJ samples. The average pH values of traditional household *doenjang* were higher than those of commercial *doenjang* [21,22], which was almost consistent with this study (average pH of HDJ and CDJ: pH 5.66 and 5.45). In fact, the acidity of traditional *doenjang* was higher than that of commercial *doenjang* samples [23]. In a previous study, during prolonged *doenjang* fermentation, organic acids and free fatty acid contents were escalated with lipolysis and amino acid biodegradation, but this has slightly deviated in this study.

In fact, 8.2–10.2% salinity was found in the HDJ samples, but a moderately higher salinity (12.8–14.6%) was recorded in the CDJ samples. The soluble protein contents were estimated at 37.54, 35.37, 25.79, and 28.79 mg/g in the 1HDJ, 2HDJ, 3HDJ, and 4HDJ samples, respectively. Besides, it was estimated that 27.73, 20.53, 20.84, and 50.83 mg/g, were in the 1CDJ, 2CDJ, 3CDJ, and 4CDJ samples, respectively. To sum up, the soluble protein contents of the HDJ samples were decently higher than that of the CDJ samples. Jeon et al. [24] reported that the salinity value (11.77–14.22%) of traditional *doenjang* showed higher than that observed (10.8–11.4%) in commercial *doenjang*. While, several studies reported that traditionally manufactured *doenjang* showed higher salinity than the commercial (modified method) *doenjang* [23,25]. However, the salinity results were remarkably higher in all types of commercial *doenjang* samples compared with the traditional *doenjang samples.* Kim et al. [26] reported that the salinity of *doenjang* differs regardless of the manufacturing method, following as the 11 traditional *doenjang* showed 8.6–14.2% and the three commercial *doenjang* showed 10.8–15.8%. Most of the previous studies reported that the salinity of traditional *doenjang* showed higher salt contents, which is used to increase the storage periods [27]. Unlike with former reports about traditional *doenjang* salinity, the salinity was found to be lower in HDJs, which might be the following reasons: HDJs were separated into liquid (*Kanjang*) and solid (*doenjang*) during fermentation, followed by the removal of salt from *doenjang* with the liquid. The liquid-separated *doenjang* was mixed with steamed soybean or *meju* powder [28].

### 2.2. Comparisons of Bacterial Distribution on HDJs and CDJs

**Figure 1:**
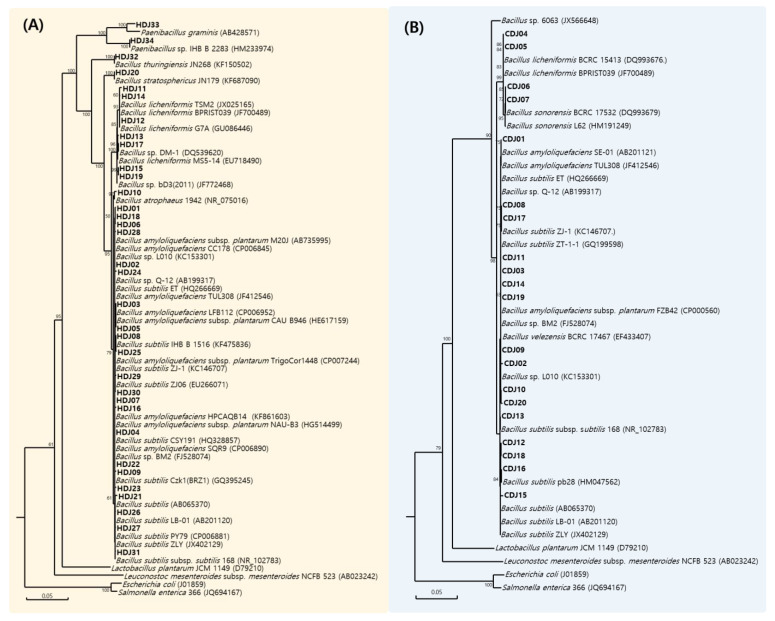
Phylogenetic placement of 16S rRNA sequences of isolates from household and commercial *doenjang*. (**A**) household *doenjang* (HDJ); and (**B**) commercial *doenjang* (CDJ). Numbers above each node are confidence levels (%) generated from 1000 bootstrap trees. The scale bar is in fixed nucleotide substitutions per sequence position.

**Figure 2:**
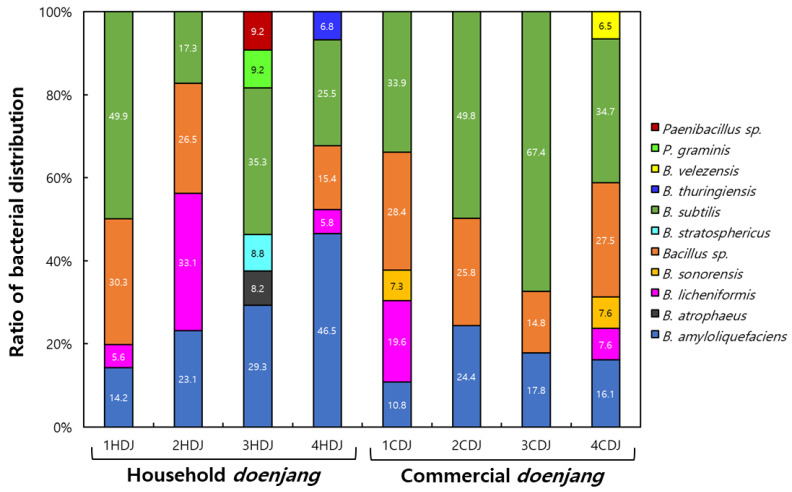
Comparisons of the bacterial distribution of isolates from household and commercial *doenjang*.

The diversity results of cultivable bacteria grown in HDJs and CDJs are shown in Table 2. Forty-three bacterial species were identified in the HDJ and CDJ samples. Among them, six *B. amyloliquefaciens*, five *B. amyloliquefaciens* subsp. *Plantarum*, one *Bacillus atrophaeus*, five *Bacillus licheniformis*, and two *Bacillus sonorensis* were found in the HDJs. Additionally, six *Bacillus* sp., one *Bacillus stratosphericus*, thirteen *Bacillus subtilis*, one *Bacillus thuringensis*, one *Bacillus velezensis*, one *Paenibacillus graminis*, and one *Paenibacillus* sp. were identified in CDJs. Based on the 16S rRNA gene sequencing, phylogenetic relationships of cultured bacteria in the HDJ and CDJ were verified (Figure 1). Because of the bacterial diversity analysis of HDJs, 34 bacteria were identified, and most of them were *Bacillus* genus except *Paenibacillus* genus (Figure 1A). Twenty bacteria were identified from the bacterial diversity analysis of CDJs (Figure 1B). All strains belonging to the *Bacillus* genus and *B. subtilis* were the most prominent enclosing the eight strains. The ratio of bacterial distribution in the HDJs and CDJs is shown in Figure 2 The major portion of bacterial strains in HDJs differed, as follows: 1HDJ—*B. subtilis* 49.9%, 2HDJ—*B. licheniformis* 33.1%, 3HDJ—*B. subtilis* 35.3%, and 4HDJ—*B. amyloliquefaciens* 46.5%. In the case of CDJs, the *B. subtilis* had the highest ratio in common (1CDJ—33.9%, 2CDJ—49.8%, 3CDJ—67.4%, and 4CDJ—34.7%).

Oh et al. [29] reported that most isolated from *doenjang* showed 99% similarity to the *Bacillus* genus, and *B. stratosphericus*, *B. altitudinis*, *B. licheniformis*, *B. aerophilus*, *B. pumilus*, *B. sonorensis*, *B. amyloliquefaciens*, *B. subtilis*, *B. lincheniformis*, and *B. mojavensis*. Regardless of salt concentrations (9%, 12%, 15%, and 18%), *Bacillus*, *Staphylococcus*, and *Clostridium* were predominant in all traditional *doenjang* samples [30]. Kim et al. [31] reported that the ten most prominent *Bacillus* species belonged to *B. sphaericus*, *B. licheniformis*, *B. pumilus*, *B. subtilis*, *B. thuringiensis*, *B. megaterium*, *B. stearothemophilus*, *B. firmus*, *B. lentus*, *B. brevis* and *Alcaligenes faecalis*, *Acinetobacter lowffii*/*junii*, and *Morganella morganii* in *doengjang*, which results were consistent with this study. In a related study, the bacterial diversity of two commercial *doenjang* was lower than that of traditional *doenjang*; *Staphylococcus gallinarum* (73.0%) and *Weissella salipiscis* (9.7%) were the main species in one type of *doenjang*, whereas *Tetragenococcus halophilus* (53.9%) and *S. gallinarum* (30.8%) were the main species in another type of *doenjang* [10]. In contrast, major bacterial species in nine traditional *doenjang* (D1-D9) varieties were observed. Particularly, sample D1 contained mainly *Enterococcus faecalis* (22.4%), *E*. *faecium* (24.2%); sample D2 comprised *B. licheniformis* (57.4%), and *B. subtilis* (21.9%); sample D3 contained *B. subtilis* (23.1%) and *B. licheniformis* (15.4%); sample D4 contained *T. halophilus* (28.2%) and *E. faecium* (20.2%); sample D5 consisted of *S. sciuri* (27.8%) and *S. lentus* (20.4%); sample D6 contained *T. halophilus* (69.8%); sample D7 contained *B. licheniformis* (26.4%) and *B. subtilis* (22.0%); sample D8 mainly contained *T. halophilus* (28.3%), and sample D9 contained *Lactobacillus halophilus* (23.7%) and *Leuconostoc mesenteroides* (15.3%). The traditional Korean *doenjang* and Japanese miso contain lactic acid bacteria such as *Enterococcus* species [32,33] that were not found in this study.

### 2.3. Comparison of Phenolic, Flavonoid, and Isoflavone Content on HDJs and CDJs

**Figure 3:**
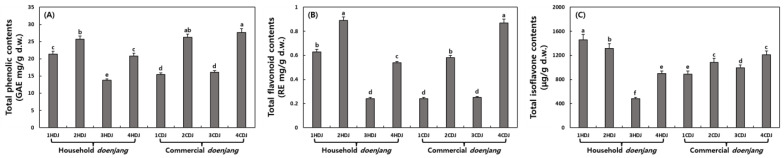
Comparison of total phenolic (**A**), total flavonoid (**B**), and total isoflavone (**C**) contents on household and commercial *doenjang*. Different letters above the bars indicate significant difference at *p* < 0.05 (*n* = 3).

**Figure 4:**
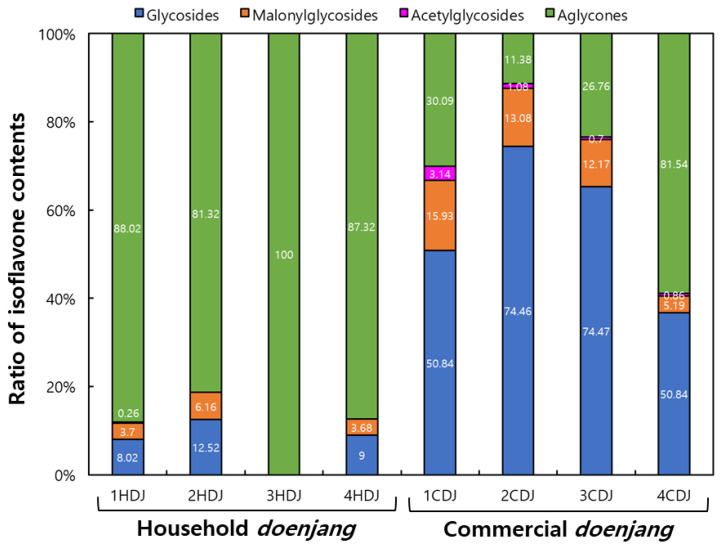
Comparisons of the four isoflavone forms (including glycoisdes, malonylglycosides, acetylglycosides, and aglycones) of 50% MeOH extracts from household and commercial *doenjang*.

The total phenolic (TP), total flavonoid (TF), and total isoflavone contents of HDJ and CDJ samples are indicated in Figure 3, Table 3, and Figure 4. The TP content of HDJ was estimated as 21.33 (1HDJ), 25.71 (2HDJ), 13.78 (3HDJ), and 20.85 (4HDJ) GAE mg/g, while the TP content of CDJs was estimated as 15.43 (1CDJ), 26.27 (2CDJ), 16.08 (3CDJ), and 27.69 (4CDJ) GAE mg/g. The HDJs and CDJs did not significantly differ in total phenolic content (Table 3). The TF contents were estimated 0.62, 0.89, 0.24, and 0.54 RE mg/g in 1HDJ, 2HDJ, 3HDJ, and 4HDJ samples, respectively. It is estimated 0.24, 0.58, 0.25, and 0.87 RE mg/g in 1CDJ, 2CDJ, 3CDJ, and 4CDJ, respectively. The TF content also did not exhibit a significant difference between HDJs and CDJs, like TP content. The total isoflavone contents were detected as 1460.80, 1314.22, 482.80, 899.38, 885.58, 1084.60, 993.84, and 1212.47 μg/g in 1HDJ, 2HDJ, 3HDJ, 4HDJ, 1CDJ, 2CDJ, 3CDJ, and 4CDJ samples, respectively. Total isoflavone content was not significantly different between HDJ and CDJ. Among the HDJ samples, two samples were confirmed to be higher than the CDJ samples, but one sample was confirmed to be the lowest among the HDJ and CDJ samples.

**Figure 5:**
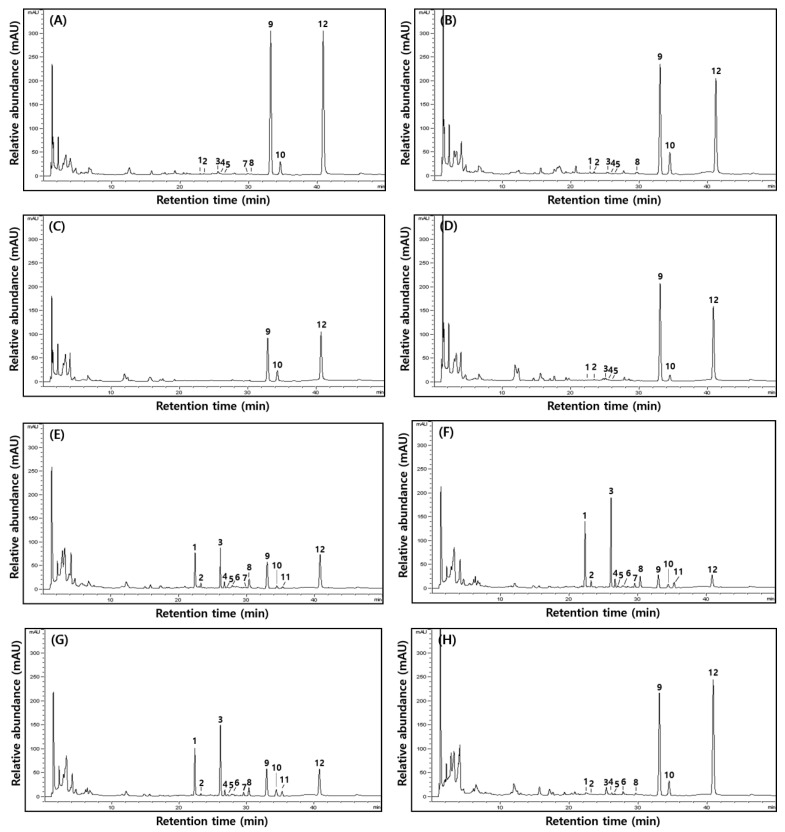
Representative HPL chromatograms of 50% MeOH extracts from household (**A**) 1HDJ, (**B**) 2HDJ, (**C**) 3HDJ, and (**D**) 4HDJ and commercial (**E**) 1CDJ, (**F**) 2CDJ, (**G**) 3CDJ, and (**H**) 4 CDJ. Note: Peaks assignment with 1, 2, 3, 4, 5, 6, 7, 8, 9, 10, 11, and 12 are marked as daidzin, glycitin, genistin, malonyldaidzin, malonylglycitin, acetyldaidzin, acetylglycitin, malonylgenistin, daidzein, glycitein, acetylgenistin, and genistein, respectively.

Glycoside, malonylglycoside, acetylglycoside, and aglycone isoflavone contents and ratio of HDJ and CDJ samples are indicated on Table 3 and Figure 4. Isoflavone glycosides and -malonylglycosides were found in 1HDJ (117.12 and 4.11 μg/g), 2HDJ (164.5080.97 μg/g), and 4HDJ (80.97 and 33.11 μg/g), except 3HDJ. Isoflavone-acetylglycosides were found only in 1HDJ (3.76 μg/g). Aglycone type isoflavone was the highest in all HDJ: 1285.81(1HDJ), 1068.75 (2HDJ), 482.80 (3HDJ), and 785.30 (4HDJ) μg/g. Notably, 3HDJ contained only aglycones. The ratio of aglycones in HDJs was more than 80% (88.02, 81.32, 100, and 87.32%), as seen in Figure 4. In contrast, all types of isoflavones were found in CDJs, where glycosides had occupied the central portion of isoflavones in 1CDJ, 2CDJ, and 3CDJ, except in 4CDJ. Notably, the aglycone content was found to be 81.54% in the 4CDJ sample. The glycoside contents in 1CDJ, 2CDJ, 3CDJ, and 4CDJ were found as 450.34, 807.65, 599.94, and 105.47 μg/g, respectively, and the aglycone content was 266.43, 123.45, 265.99, and 988.60 μg/g, respectively. Consequently, glycoside content was highest in 2CDJ (807.65 μg/g), and aglycone content was highest in 4CDJ (988.60 μg/g). To sum up, the glycoside was highest in 2CDJ (74.46%), and aglycone was highest in 4CDJ (81.54%), the overall isoflavone ratio in CDJs. Figure 5 shows a typical HPLC chromatogram of 50% ethanol *doenjang* extracts. In the case of 3HDJ, only isoflavone aglycone forms, such as daidzein, glycitein, and genistein, were found.

Shukla et al. [21] reported that no individual differences in TP content were observed in *doenjang* made with *meju* and commercial *doenjang*. Ahn et al. [34] reported that the TP content of traditional *doenjang* and commercial *doenjang* ranged from 18.71–25.78 GAE mg/mL. Most traditional *doenjang* is usually made using soybeans, salt, and water, whereas commercial *doenjang* components are koji, soybean, wheat, barley, salt, and water. Therefore, TP content among doenjang types differs according to *doenjang* composition material [17,18]. Kim et al. [25] reported that the major isoflavone of traditional *doenjang* was daidzein and genistein. Generally, isoflavones in soybean are found in glycosides form, e.g., genistin, daidzin, glycitin, which are converted into aglycone e.g., genistein, daidzein, glycitein by β-glucosidase activities of microorganisms [35,36,37]. In addition, Kwak et al. [38] reported that the aglycone contents increased with the progress of fermentation periods of *doenjang*. In fact, the household *doenjang* fermentation time was longer than the commercial *doenjang*. It is supposed that the high aglycone ratio and content in HDJs were because of the longer fermentation period, while the high glycoside ratio and content in CDJs were due to the short fermentation period. These results lead us to conclude that the distribution of glycosides and aglycone would be an indicator to estimate the approximate fermentation period of *doenjang*.

### 2.4. Comparison of Antioxidant Activities on HDJs and CDJs

**Figure 6:**
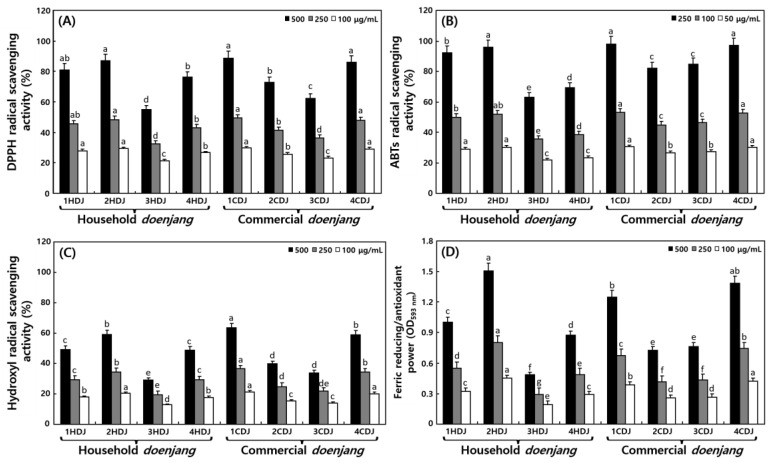
Comparisons of antioxidant activities of 50% MeOH extracts from household and commercial *doenjang*. (**A**) DPPH radical scavenging activity; (**B**) ABTS radical scavenging activity; (**C**) hydroxyl radical scavenging activity; and (**D**) ferric reducing/antioxidant power. All values are means of determination from three independent experiments. Different letters above the bars indicate significant difference at *p* < 0.05.

The DPPH, ABTS, and hydroxyl radical scavenging activities and FRAP of HDJ and CDJ are shown in Figure 6. Interestingly, no significant differences in DPPH radical scavenging activities between HDJ and CDJ samples were found. In the case of HDJ, the highest DPPH radical scavenging activity was found in 2HDJ samples while it was obtained maximum in 1CDJ among other CDJ samples (Figure 6A). Notably, at 100, 250, and 500 μg/mL of sample concentrations, the 2HDJ showed maximum 29.23%, 48.46%, and 86.92% DPPH radical scavenging activities, respectively. Similarly, the 1CDJ demonstrated the highest 29.68%, 49.36%, and 88.73% DPPH activities, respectively. Among these *doenjang* samples, the highest DPPH activities were provided by the 1CDJ samples in this study (Figure 6A).

Additionally, the 2HDJ and 1CDJ showed the highest ABTS radical scavenging activities among the HDJ and CDJ samples, respectively (Figure 6B). At 50, 100, and 250 μg/mL sample concentration, the 2HDJ showed the highest 29.99%, 51.97%, and 95.95% among others HDJ, while 1CDJ showed maximum 30.49%, 27.14%, and 97.94%, ABTS radical scavenging activities, respectively. However, the 3HDJ showed the lowest, and 1CDJ showed the highest ABTS radical scavenging activity among all other *doenjang* samples (Figure 6B).

Not many significant differences in hydroxyl radical scavenging activities between the HDJ and CDJ samples were observed (Figure 6C), as it was pronounced for DPPH and ABTS radical scavenging activities. In fact, the highest hydroxyl radical scavenging activity was expressed by the 2HDJ and 2CDJ samples, among other HDJ and CDJ, respectively. At 100, 250, and 500 μg/mL of sample concentration, the 2HDJ showed 20.26%, 34.51%, and 59.02%, whereas 1CDJ expressed 21.31%, 36.61%, and 63.22%, hydroxyl radical scavenging activities, respectively. At 500 μg/mL sample, the lowest activity obtained 28.97% in 3HDJ, whereas the highest activity was 63.22% in 2CDJ (Figure 6C).

The FRAP of HDJs and CDJs was assessed at 100, 250 and 500 μg/mL sample. Among HDJs, the 2HDJ demonstrated the highest activity with decreasing power of 0.45, 0.80, and 1.51 at 100, 250, and 500 μg/mL sample treatments, respectively. Notably, the 3HDJ had the lowest reducing power of 0.20, 0.29, and 0.49, respectively (Figure 6D).

The fermentation process increased the antioxidant activity of fermented soybean foods, such as tofu, *doenjang*, and soymilk [39,40]. Generally, the fermentation period of household *doenjang* was longer than that of the commercial *doenjang* [18], but the radical scavenging activities of the HDJs was not significantly greater than that of the CDJs. It is judged to occur due to the decrease in phenolic contents in the manufacturing environment of HDJs compared to the CDJs. Similar concentrations of the samples showed higher activity in ABTS than in DPPH. This was observed because DPPH reacts with free radicals, but ABTS reacts with different free radicals, including peroxyl, hydroxyl, alkoxyl, and inorganic radicals, to form stable ABTS^+^ type cationic radicals; due to having these differences, a difference in binding capacity to antioxidants occurred [41]. Therefore, ABTS^+^ measured the antioxidant power of hydrophilic and hydrophobic substances, so it was judged to exhibit higher scavenging activity compared to DPPH [42]. The hydroxyl radical scavenging activity of fermented soybean yogurt and fermented soybean solid was estimated at 39% (500 μg/mL) and 49% (200 μg/mL) [43,44], which indicated a higher amount compared to *doenjang* used in the study. According to the report by Lee et al. [7], radical scavenging activity and FRAP were significantly connected with aglycone and phenolic compounds. However, Shukla et al. [21] reported that *doenjang*’s FRAP activity was slightly crosslinked with phenolic compounds, which agreed with our study. Subsequently, aglycones, phenolics, and flavonoids influenced antioxidant activities without showing any consistent patterns. Previous studies reported that isoflavone aglycone and phenolic compound content influenced antioxidant activity [7,45,46]. However, phenolics, flavonoids, and isoflavones did not consistently demonstrate a pattern with antioxidant activity, like DPPH, ABTS, FRAP reducing power, and hydroxyl radical scavenging activities between HDJs and CDJs. However, when they were compared and analyzed comprehensively, samples with high phytochemicals demonstrated high antioxidant activity.

### 2.5. Comparison of DNA Damage Protecting Activity on HDJs and CDJs

**Figure 7:**
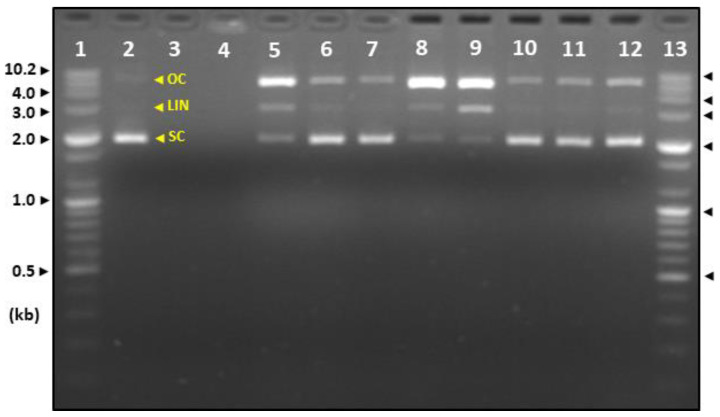
Comparisons of agarose gel electrophoresis for DNA protective effects of 50% MeOH extracts from household and commercial *doenjang*. Lane 1 and 13, size mark; lane 2, untreated; lane 3, blank; lane 4, treated; lane 5–8, 1HDJ–4HDJ; and lane 9–12, 1CDJ–4CDJ. OC, open circular; LIN, linear; and SC, supercoiled.

The DNA damage protecting activity of HDJs and CDJs against oxidative stress (Fenton’s reagent) is shown in Figure 7. In this study, Fenton’s reagent was used as an oxidizing agent to verify the OC (Open Circular DNA), LIN (Linear DNA), and SC (Supercoil DNA) protecting activity of HDJs and CDJ samples. The treatment of Fenton’s reagent to plasmid DNA showed OC, LIN, and SC at about 8 kb, 3 kb, and 2 kb, respectively, in the untreated positive control. In the case of the negative control, Fenton’s reagent treatment caused the damage to all three DNAs damaged. Consequently, the DNA could not be identified as shown in line 4 (Figure 7). All OC, LIN, and SC DNA were defined in 1HDJ, 2HDJ, 3HDJ, and 4HDJ, but 1HDJ and 4HDJ demonstrated the strongest OC DNA band, followed by LIN and SC. Besides, 2HDJ and 3HDJ demonstrated the DNA protective effect of the same band pattern as the positive control. DNA protecting activity of 1CDJ, 2CDJ, 3CDJ, and 4CDJ also verified the OC, LIN, and SC DNA and household *doenjang*. Among them, 1CDJ demonstrated the strongest OC DNA band, followed by LIN and SC, and 2CDJ, 3CDJ, and 4CDJ exhibited the same DNA protective effect as a positive control.

Boukhris et al. [47] reported that flower extract caused no damage to DNA. Still, the bands’ fluorescence intensity was altered because the bands of supercoil DNA weakened, and the open circle DNA became stronger when DNA damage occurred. Gao et al. [48] reported that the plasmid DNA is used to identify DNA damage as it is found in the form of supercoil. It was converted into a gentle open circular DNA and linear DNA form when H_2_O_2_ and Fe^2+^ were added. It also validated the highest DNA protective effect at a concentration of 200 mol/L of *Shallercapus gracilis* extract. Akher et al. [49] validated the high protecting activity of the supercoil DNA bands in extracts with ethanol and methanol of *Boerhaavia diffusa*, similar to the control. Additionally, it was reported that the DNA protective effect was low in samples where the supercoil DNA band’s fluorescence intensity was reduced, and the circular DNA band’s fluorescence intensity was increased. When plasmid DNA damage occurred, a phosphate ester bond of the supercoil DNA was cleaved to produce relaxed open circular DNA, and the portion near the DNA where the first crack occurred was cleaved and changed to linear double-stranded DNA [47,50,51]. In this regard, when the protecting activity against DNA damage is verified, the damage to supercoil DNA occurs the least. It was judged that the material with low open circular DNA and linear DNA formation has a high DNA protective effect. Therefore, 2HDJ, 3HDJ, 2CDJ, 3CDJ, and 4CDJ exhibited a similar DNA protective effect as the control without Fenton’s reagent. Additionally, Marazza et al. [52] reported that as the soy milk fermented, DNA protective effect increased, and reported that the greatest DNA protective effect was shown at 24 h of fermentation. It was reported that this was because of the isoflavone aglycone contents, which varied from these results. In this regard, it was judged that it is necessary to verify the relationship with different compounds in addition to aglycones. These studies on the DNA protective effect of soybean and fermented foods using soybean for the Fenton reaction have been insignificant. Therefore, this study verifies the DNA protective effect of *doenjang* on the Fenton reaction and will be used as basic data for studying the DNA protection effect of soybean and soybean-fermented food.

## 3. Materials and Methods

### 3.1. Medium and Chemical

The LB broth and agar media were purchased from Difco (Becton Dickinson Co., Spark, MD, USA). In order to measure the antioxidants and the enzyme activities, the reagents 2,2-diphenyl-1-picrydrazyl (DPPH), 2,4,6-azino-bis (3-ethylbenzthiazoline-6-sulfonic acid) (ABTS) and 2,4,5-tri(2-pyridyl)-1,3,5-triazine (TPTZ) were purchased from Sigma-Aldrich Co. (St, Louis, MO, USA). Chemicals for measuring TP and TF contents (such as the Folin-Ciocalteu’s reagent and diethylene glycol) were also purchased from Sigma-Aldrich. Amongst the twelve isoflavone standards, daidzin, glycitin, genistin, daidzein, glycitein, genistein, malonyldaidzin, malonylglycitin, malonylgenistin, acetyldadzin, acetylglycitin, and acetyldaidzin were purchased from Sigma-Aldrich and the LC Laboratories (Woburn, MA, USA). For the analysis, the reagents and solvents (such as HPLC-grade water, methanol, acetonitrile, glacial acetic acid, etc.) were purchased from Sigma-Aldrich and Fisher Scientific International, Inc. (Fairlawn, NJ, USA), respectively.

### 3.2. Collection of Doenjang Samples

**Figure 8:**
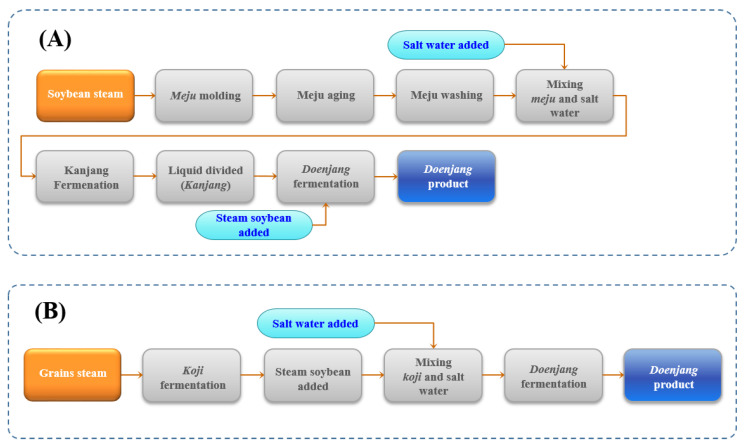
Manufacturing process of household (**A**) and commercial (**B**) *doenjang*.

The standard manufacturing process of household *doenjang* and commercial *doenjang* is shown in Figure 8. Traditional household *doenjang* is generally prepared at home in Gyeongnam province for a long time. Four household *doenjang* samples (1HDJ: household *doenjang* sample No.1, 2HDJ: household *doenjang* sample No.2, 3HDJ: household *doenjang* sample No.3 and 2HDJ household *doenjang* sample No.4) were obtained from homes in Jinju, Hadong, Hamyang, and Sancheong cities of Gyeongnam–do province. Additionally, four commercial *doenjang* samples (1CDJ: commercial *doenjang* sample No.1, 2CDJ, commercial *doenjang* sample No.2, 3CDJ: commercial *doenjang* sample No.3 and 4CDJ: commercial *doenjang* sample No.4) were bought from different manufacturers in South Korea.

### 3.3. Determination of pH, Acidity, Salinity and Soluble Protein Content on Doenjang

The *doenjang* (10 g) was mixed with 90 mL distilled water and shaken for 30 min. Next, the supernatant was obtained by centrifugation (13,000× *g*, 3 min). The supernatant’s pH was measured using a pH meter (MP 220 pH meter, Schwerzenbach, UK), and the acidity (lactic acid, %) was measured by titrating the supernatant with 0.1 NaOH until pH was 8.2 ± 0.1. For salinity measurements, 10 g *doenjang* was mixed with 40 mL distilled water, and the supernatant was obtained after shaking and centrifugation, as stated above. The supernatant’s salinity was measured using a salt meter (PAL-03S, ATAGO, Japan).

The soluble protein contents were measured using the biuret method [53]. Four milliliters of biuret reagent and 1 mL *doenjang* sample extract were placed in a screw-capped test tube and kept in a hot water bath at 37 °C for 20 min. The absorbance was measured at 540 nm using a spectrophotometer (Thermo Scientific GENESYS 20 Spectrophotometer, CA, USA). The crude protein concentration was measured using a bovine serum albumin standard curve.

### 3.4. Microbial Isolation and Identification

Eight *doenjang* samples were diluted with sterile physiological saline. The diluted samples (0.1 mL) were spread on tryptic soy agar plates, and incubated for 48 h at 37 °C, and after the generated colonies were measured. Each experiment was repeated three times as an average value and expressed as colony forming unit (log cfu/g) per gram of sample.

Selected colonies were cultivated in LB broth for 12 h with shaking (150 rpm) at 37 °C. The culture (3 mL) was centrifuged in sterile tube at 13,000× *g* for 3 min. Total DNA were isolated from the collected cells using G-spin genomic DNA Purification Kit (Intron Biotechnology, Suwon, Korea). The 16S rRNA gene sequences were confirmed as described by Cho et al. [45]. The determined 16S rRNA gene sequence was compared with another bacterial 16S rRNA obtained from the GeneBank database. The similarity values were calculated from alignments, evolutionary distances using a DNAMAN analysis system (Lynmon Biosoft, Quebec, Canada). Phylogenetic trees were identified using the neighbor-joining method and distance matrix data.

### 3.5. Preparation of Doenjang Extracts

One gram of freeze-dried powder sample was mixed with 50% methanol (20 mL) and shaken for 12 ± 2 h at room temperature. After that, the mixture was centrifuged, followed by extracting the supernatant with a syringe filter (diameter 25 mm, pore size 0.45 μm, Chromdisc, Maidstone, UK).

### 3.6. Determeination of the TP and TF Contents of Doenjang Extracts

The TP contents were determined using the Folin Denis method [54]. A total of 0.5 mL diluted extract was dispensed into a test tube, and a 0.5 mL 25% Na_2_CO_3_ solution was added and kept for 3 min. Next, 0.25 mL of 2 N Folin Ciocalteu phenol reagent was added to the reaction solution and kept at 30 °C for 1 h. After that, the solution absorbance was measured at 750 nm. The total phenolic contents were measured as per the gallic acid standard curve.

The TF contents was determined by the method as described Lee et al. [55]. Briefly, 0.5 mL of diluted extract was dispensed into a test tube, and 1 mL of diethylene glycol and 0.01 mL of 1 N NaOH were added and reacted in a hot water bath at 37 °C for 1 h. The absorbance was measured at 420 nm using a spectrophotometer and the total flavonoid contents were measured as rutin standard curve.

### 3.7. HPLC Analysis of the Isoflavone Content of Doenjang Extracts

The isoflavone analysis was conducted by HPLC (Agilent 1200 series, Agilent Co. Forest Hill, VIC, Australia), according to Hwang et al. [43]. The Lichrophore 100 RP C18 column (4.6 × 250 mm, 5 μm, Merck, Germany) was set toward mobile phase 0.2% glacial acetic acid in water (solution A) and 100% acetonitrile (solution B). The analysis conditions were set to 100%/0 min, 90%/15 min, 80%/25 min, 75%/30 min, 65%/45 min, and 65%/50 min with solvent A. Finally, 20 μL each sample was injected into the column, and the solvent flow rate was set to 1 mL/min at 30 °C. A detector was used as a diode array detector (Agilent 1200 series, Agilent Co.) and quantified at 254 nm absorbance.

### 3.8. Antioxidant Activities of Doenjang Extracts

#### 3.8.1. DPPH Radical Scavenging Activity

The DPPH radical scavenging activity of each extract was determined using Lee et al. [54] method. In addition, 0.2 mL of each sample extract was mixed with 0.8 mL 1.5 × 10^−4^ M DPPH reagent in ethanol. The mixture was incubated for 30 min at room temperature in the dark, and their absorbance was measured at 525 nm using a spectrophotometer. The extract solution was used as a negative control. The DPPH radical scavenging effects were calculated using the following equation:

(1) $$Radical\;scavenging\;activity\left(\%\right)=[1-(Negative\;control÷Sample)]\times 100$$

#### 3.8.2. ABTS Radical Scavenging Activity

The ABTS radical scavenging activity of each extract was measured according to Lee et al. [54]. The ABTS solution (a mixture of 5 mL 7 mM ABTS and 5 mL 2.45 mM K_2_S_2_O_8_) was kept in the dark at room temperature for 12–16 h. After that, the solution was diluted using methanol to adjust its absorbance (OD) to around 0.7 ± 0.02 at 734 nm wavelength. Next, 0.1 mL each sample extracts were allowed to react with 0.9 mL ABTS solution for three minutes. Finally, the absorbance of the solutions was measured at a 732 nm wavelength in a spectrophotometer. The unreacted extract solution was used as a negative control. The ABTS radical scavenging effects were calculated using the following Equation (1).

#### 3.8.3. Hydroxyl Radical Scavenging Activity

The hydroxyl radical scavenging activity of each *doenjang* extract was determined using Adjimani and Asare’s [56] method. Briefly, 1.4 mL sample extracts were mixed with 0.2 mL 10 mM FeSO_4_.7H_2_O-EDTA, 0.2 mL 10 mM 2-deoxyribose, and 0.2 mL 10 mM H_2_O_2,_ consequently, the mixture was incubated at 37 °C for 4 h. Thenceforth, 1 mL 1% thiobarbituric acid and 1 mL 2.8% trichloroacetic acid were added to the reaction mixture and sunk in a hot water bath at 100 °C for 20 min. Next, the reactant mixture was cooled to room temperature to measure absorbance at 520 nm wavelength in a spectrophotometer. The phosphate buffer saline was used as a negative control during the absorbance measurement of the reactant samples. The hydroxyl radical scavenging effects were calculated using the following Equation (1).

#### 3.8.4. Ferric Reducing/Antioxidant Power

The ferric reducing/antioxidant power (FRAP) of each *doenjang* extract was assayed as per the modified method of Wen et al. [57]. The FRAP reagent was mixed with acetate buffer (30 mM, pH 3.6), TPTZ reagent (10 mM in 40 mM HCl), and FeCl_3_ solution (20 mM in DW) in a ratio of 10:1:1 (*v*/*v*/*v*). Next, the reaction mixture was allowed to react at 37 °C for 15 min in a water bath. Later, 0.95 mL FRAP reagent mixture was added with 0.05 mL *doenjang* extracts and incubated at 37 °C for 15 min. Finally, the solution absorbance was measured at 593 nm using a spectrophotometer.

### 3.9. DNA Damage Protecting Activities of Doenjang Extracts

DNA damage protecting activity of each *doenjang* extract was determined against SK+ vector plasmid DNA. Briefly, *doenjang* sample extracts, SK+ vector plasmid DNA, 1× TAE buffer (10 mM Tris-HCl and 1 mM EDTA), and Fenton’s reagents (100 mM H_2_O_2_, 0.1 mM acetic acid and 1.6 mM FeCl_3_) were prepared in advance. A negative control reaction mixture was made of 2 μL DNA (SK+ vector) and 18 μL sterile water. While the positive control reaction mixture comprised 2 μL DNA (SK+ vector), 10 μL *doenjang* sample extracts, and 8 μL Fenton’s reagent. The experiment reaction mixture consisted of 2 μL DNA, 10 μL *doenjang* sample extract, and 8 μL Fenton’s reagent. The reaction mixture was kept at 30 °C for 1 h in a water bath. After each reaction, the agarose gel (1.5%) electrophoresis was conducted to confirm the DNA degradation protection capabilities of the *doenjang* sample extracts [52].

### 3.10. Statistical Analyses of Nutritional Compositions and Antioxidant Properties

All determination values were based on three independent experiments with triplicate samples (*n* = 3). The results of nutritional component contents and antioxidant ratios in samples were represented as the mean ± SD values. Significant differences between samples were documented by Duncan’s multiple range test (*p* < 0.05) through ANOVA procedure using the Statistical analysis system (SAS) software (ver. 9.4; SAS institute, Cary, NC, USA).

## 4. Conclusions

This study reported for the first time a variation in physicochemical features, antioxidant activities, secondary metabolites (TP, TF and isoflavone), cultivable bacterial diversities, and DNA protective effects of household and commercial *doenjang*. Characteristically, HDJ samples showed larger aglycone content ratios and greater diversity of bacterial distribution. In contrast, CDJ samples verified higher glycoside content ratios and relatively simple bacterial distribution. In particular, the greater ratios of bacterial strains were recorded as 49.9% *B. subtilis* in 1HDJ, 33.1% *B. licheniformis* in 2HDJ, 35.3% *B. subtilis* in 3HDJ, and 46.5% *B. amyloliquefaciens in* 4HDJ samples, while *B. subtilis* had the highest ratio 33.9–49.8% in common in CDJ samples. Therefore, it suggests that the greater portion of aglycone contents generation in HDJs were associated with bacterial diversity. However, the amounts of TP, TF, and isoflavone contents, antioxidant activities, and DNA protection effects were variedly observed regardless of HDJs and CDJs. These are estimated to be due to different manufacturing techniques included fermentation conditions, raw material content, soybean varieties, and cultivation environments (climate, temperature, and region). Due to enclosing higher aglycone contents ratios in HDJ samples, it is better to focus on how these biofunctional contents can be increased in CDJ production. Moreover, further studies should be conducted on the fermentation conditions, raw material, and enrichment of phytochemicals—in HDJs and CDJs.

## Figures and Tables

**Table 1:** Comparisons of physicochemical properties of household and commercial *doenjang*.

Samples ^1^	pH	Acidity (%, as Lactic Acid)	Salinity (%)	Soluble Protein Contents (mg/g)
1HDJ	5.71 ± 0.29a ^2^	1.41 ± 0.04f	9.00 ± 0.54d	37.54 ± 1.13b
2HDJ	5.69 ± 0.28a	1.36 ± 0.05f	10.20 ± 0.61c	35.37 ± 1.05b
3HDJ	5.31 ± 0.27b	1.86 ± 0.06d	8.20 ± 0.49e	25.69 ± 0.75d
4HDJ	5.94 ± 0.30a	3.03 ± 0.09a	10.00 ± 0.60c	28.79 ± 0.80c
1CDJ	5.37 ± 0.27b	2.21 ± 0.07c	14.20 ± 0.71a	27.73 ± 0.83c
2CDJ	5.64 ± 0.25ab	2.87 ± 0.09b	12.80 ± 0.58b	20.53 ± 0.60e
3CDJ	5.64 ± 0.34ab	1.61 ± 0.06e	12.80 ± 0.77b	20.84 ± 0.62e
4CDJ	5.14 ± 0.26c	1.59 ± 0.05e	14.60 ± 0.88a	50.83 ± 1.52a

^1^ HDJ, household *doenjang*; CDJ, commercial *doenjang*. ^2^ All values are presented as the mean ± SD of three independent experiments. Different letters correspond to the significant difference using Duncan’s multiple test (*p* < 0.05).

**Table 2:** Comparisons of 16S rRNA sequence similarity and viable cell numbers of isolates from household and commercial *doenjang*.

Isolates	Nearest Relative (Accession No.)	Similarity ^1^ (%)	Log cfu/g							
			1HDJ	^2^ 2HDJ	3HDJ	4HDJ	1CDJ	2CDJ	3CDJ	4CDJ
DJ01	*Bacillus amyloliquefaciens* CC178 (CP006845)	99–100				10.01				
DJ02	*Bacillus amyloliquefaciens* HPCAQB14 (KF861603)	99		9.36	5.52	8.94				
DJ03	*Bacillus amyloliquefaciens* LFB112 (CP006952)	100			4.78					
DJ04	*Bacillus amyloliquefaciens* SE-01 (AB201121)	99						4.04		
DJ05	*Bacillus amyloliquefaciens* SQR9 (CP006890)	99				7.0				
DJ06	*Bacillus amyloliquefaciens* subsp. *Plantarum* CAU B946 (HE617159)	99		6.0						
DJ07	*Bacillus amyloliquefaciens* subsp. *Plantarum* M20J (AB735995)	99	5.0							
DJ08	*Bacillus amyloliquefaciens* subsp. *Plantarum* NAU-B3 (HG514499)	99				8.39				
DJ09	*Bacillus amyloliquefaciens* subsp. *Plantarum* FZB42 (CP000560)	99								5.30
DJ10	*Bacillus amyloliquefaciens* subsp. *Plantarum* TrigoCor1448 (CP007244)	99				6.0				
DJ11	*Bacillus amyloliquefaciens* TUL308 (JF412546)	99	7.61	8.05	5.73	8.0	5.89	4.93	4.93	5.30
DJ12	*Bacillus atrophaeus* 1942 (NR_075016)	99			4.48					
DJ13	*Bacillus licheniformis* BCRC 15413 (DQ993676)	99					4.90			
DJ14	*Bacillus licheniformis* BPRIST039 (JF700489)	99		8.0		6.0	5.85			5.0
DJ15	*Bacillus licheniformis* G7A (GU086446)	99		8.30						
DJ16	*Bacillus licheniformis* MS5-14 (EU718490)	99	5.0	8.0						
DJ17	*Bacillus licheniformis* TSM2 (JX025165)	99		9.36						
DJ18	*Bacillus sonorensis* BCRC 17532 (DQ993679)	99								5.0
DJ19	*Bacillus sonorensis* L62 (HM191249)	99					4.0			
DJ20	*Bacillus* sp. 6063 (JX566648)	99								5.0
DJ21	*Bacillus* sp. bD3(2011) (JF772468)	99	8.0	8.0						
DJ22	*Bacillus* sp. BM2 (FJ528074)	99–100	7.60	8.0			5.72	4.70	4.11	
DJ23	*Bacillus* sp. DM-1 (DQ539620)	99	6.30			8.02				
DJ24	*Bacillus* sp. L010 (KC153301)	99	5.0	5.0			4.00	4.78		6.58
DJ25	*Bacillus* sp. Q-12 (AB199317)	99		6.0		8.02	5.85			6.57
DJ26	*Bacillus stratosphericus* JN179 (KF687090)	99			4.78					
DJ27	*Bacillus subtilis* (AB065370)	99	5.0					4.60	5.03	
DJ28	*Bacillus subtilis* CSY191 (HQ328857)	99	5.30	9.34						
DJ29	*Bacillus subtilis* Czk1(BRZ1) (GQ395245)	99			5.11					
DJ30	*Bacillus subtilis* ET (HQ266669)	99	7.0				4.85			5.30
DJ31	*Bacillus subtilis* IHB B 1516 (KF475836)	99	7.0							
DJ32	*Bacillus subtilis* LB-01 (AB201120)	99			4.0		5.04		4.78	5.30
DJ33	*Bacillus subtilis* pb28 (HM047562)	99						4.48		
DJ34	*Bacillus subtilis* PY79 (CP006881)	99				8.09				
DJ35	*Bacillus subtilis* subsp. *Subtilis* 168 (NR_102783)	99	7.41					4.30		
DJ36	*Bacillus subtilis* ZJ06 (EU266071)	99				9.68				
DJ37	*Bacillus subtilis* ZJ-1 (KC146707)	99–100	6.34	8.30	5.72	8.74		4.81	5.0	6.11
DJ38	*Bacillus subtilis* ZLY (JX402129)	99	6.30		4.48		4.70		3.90	6.26
DJ39	*Bacillus subtilis* ZT-1-1 (GQ199598)	99					4.0			
DJ40	*Bacillus thuringiensis* JN268 (KF150502)	100				7.08				
DJ41	*Bacillus velezensis* BCRC 17467 (EF433407)	99								4.30
DJ42	*Paenibacillus graminis* (AB428571)	98			5.0					
DJ43	*Paenibacillus* sp. IHB B 2283 (HM233974)	99			5.0					

^1^ Range of 16S rRNA gene sequences is similarity values between reference strain and isolated strain from homemade and commercial *doenjang* samples. ^2^ HDJ, household *doenjang*; CDJ, commercial *doenjang*.

**Table 3:** Comparison of glycoside, malonylglycoside, acetylglycoside, and aglycone isoflavone contents on household and commercial *doenjang*.

Contents ^1^	Samples							
	1HDJ	2HDJ	3HDJ	4HDJ	1CDJ	2CDJ	3CDJ	4CDJ
**Glycosides**								
Daidzin	43.62 ± 2.62f	73.39 ± 4.40d	nd ^2^	38.78 ± 1.75g	209.62 ± 12.58c	338.48 ± 20.31a	265.53 ± 13.28b	62.08 ± 3.11e
Glycitin	44.42 ± 2.67d	54.58 ± 3.27c	nd	28.05 ± 1.26f	78.71 ± 4.72b	100.24 ± 6.01a	45.28 ± 2.26d	42.68 ± 2.13e
Genistin	29.08 ± 1.74f	36.53 ± 2.19e	nd	14.14 ± 0.64g	162.01 ± 9.72c	368.93 ± 22.14a	289.13 ± 14.46b	45.71 ± 2.29d
Total	117.12	164.50	nd	80.97	450.34	807.65	599.94	150.47
**Malonylglycosides**								
Daidzin	3.72 ± 0.22g	6.75 ± 0.41f	nd	13.51 ± 0.61d	32.38 ± 1.94b	43.61 ±2.62a	28.30 ±1.42c	8.31 ±0.42e
Glycitin	25.16 ± 1.51b	24.83 ± 1.49b	nd	19.60 ± 0.88c	24.22 ± 1.45b	27.84 ±1.67a	24.64 ±1.23b	24.06 ±1.20c
Genistin	25.23 ± 1.51f	49.39 ± 2.96d	nd	nd	84.43 ± 5.07a	70.46 ±4.23b	68.00 ±3.40c	30.55 ±1.53e
Total	54.11	80.97	nd	33.11	141.03	141.91	120.94	62.92
**Acetylglycosides**								
Daidzin	nd	nd	nd	nd	24.99 ± 1.50a	9.61 ± 0.58c	5.60 ± 0.28d	10.48 ± 0.52b
Glycitin	3.76 ± 0.23a	nd	nd	nd	1.86 ± 0.11b	1.32 ± 0.08c	0.91 ± 0.05d	nd
Genistin	nd	nd	nd	nd	0.93 ± 0.06a	0.66 ± 0.04b	0.46 ± 0.02c	nd
Total	3.76	nd	nd	nd	27.78	11.59	6.97	10.48
**Aglycones**								
Daidzein	686.58 ± 41.19a	548.20 ± 32.89b	200.50 ± 9.02c	467.55 ± 21.04b	123.42 ± 7.41d	52.22 ± 3.13e	124.90 ± 6.25d	485.39 ± 24.27b
Glycitein	98.44 ± 5.91b	162.60 ± 9.76a	72.55 ± 3.26c	47.08 ± 2.12d	19.67 ± 1.18g	23.70 ± 1.42f	42.45 ± 2.12e	102.31 ±5.12b
Genistein	500.79 ± 30.05a	357.95 ± 21.48c	209.75 ± 9.44e	270.67 ± 12.18d	123.34 ± 7.40f	47.53 ± 2.85h	98.64 ± 4.93g	400.90 ± 20.05b
Total	1285.8	1068.75	482.80	785.30	266.43	123.45	265.99	988.60

^1^ All values are presented as the mean ± SD of three independent experiments. Different letters correspond to the significant difference using Duncan’s multiple test (*p* < 0.05). ^2^ nd: not detected.

## Data Availability

The data that support the findings of this study are available from the corresponding author, upon reasonable request.

## Sample Availability

Samples of the compounds are available from the authors.
